# Detection and functional analysis of horizontal gene transfer events in the ciliate *Euplotes*

**DOI:** 10.3389/fmicb.2026.1782463

**Published:** 2026-04-08

**Authors:** Ruanlin Wang, Xuwei Wang, Qingyao Meng, Xiaoyan Liu, Haiqing Yan, Zhiyun Zhang, Yuejun Fu, Aihua Liang

**Affiliations:** Key Laboratory of Chemical Biology and Molecular Engineering of Ministry of Education, Institute of Biotechnology, Shanxi University, Taiyuan, China

**Keywords:** ciliate, *Euplotes*, horizontal gene transfer, mannosidase, symbiosis

## Abstract

**Background:**

Horizontal gene transfer (HGT), the movement of heritable materials between distantly related organisms, is a key evolutionary force shaping eukaryotic genomes. *Euplotes* are free-living unicellular eukaryotes belonging to the phylum Ciliophora, and are tended to establish endosymbiotic relationships with different bacteria. However, the scale of HGT in *Euplotes*, and its possible roles in driving their diversification and adaptation remains unexplored.

**Methods:**

A large-scale phylogeny-based bacterial HGT detection was performed across five genome sequenced *Euplotes*. Gene structure and expression of the HGT-acquired genes were analyzed based on the transcriptome data. Putative functions of these genes were annotated based on BLAST search in the protein family (Pfam), the Gene Ontology (GO) and the Kyoto Encyclopedia of Genes and Genomes (KEGG) database. Quantitative polymerase chain reaction (qPCR) and RNA interference (RNAi) were performed to validate the function of the prevalent HGT-acquired genes encoding mannan endo-1,4-β-mannosidase (Man) from *E. amieti*.

**Results:**

We systematically examined HGT in five *Euplotes* genomes and found that they acquired a total of 342 genes exhibiting diverse functions, including enzymes involved in carbohydrate metabolism, sulfur metabolism, and the cell signaling. HGT-acquired genes displayed similar genomic features with the native genes, including GC content, the proportion of intron-contained gene, and coding sequences (CDS) length, implying ancient acquisition events. Five putative endosymbiont-derived genes encoding glycoside hydrolases from *E. vannus* were identified. Furthermore, among the 342 HGT candidates, only seven HGT families were putatively transferred into the last common ancestor of all five *Euplotes*. Further qPCR analysis showed that the mRNA levels of mannan endo-1,4-β-mannosidase A (*Ea-ManA*) and mannan endo-1,4-β-mannosidase B (*Ea-ManB*) increased after feeding with *Chlorogonium elongatum* in *E. amieti*. Knockdown of *Ea-Man* genes by RNAi increased mortality which suggested that *Ea-Man* genes are essential for *E. amieti*.

**Conclusion:**

Based on these findings, we suggest that the endosymbionts of *Euplotes* are potential donor organisms for HGT-acquired genes, and HGT is a prevalent mechanism that is actively used in *Euplotes* to expand their adaptive capabilities.

## 1 Introduction

The genetic information of an organism is usually transmitted vertically from parent to progeny. However, organisms can also acquire genes from foreign sources via horizontal gene transfer (HGT) (Soucy et al., 2015). HGT refers to the transfer of genetic material between different species through non-reproductive means, in contrast to vertical gene transfer, which occurs from parent to progeny. The process has long been understood to be a major driver of genome evolution (Smith et al., 1992; Ochman et al., 2000). Although HGT is prevalent and well documented in bacteria and archaea (Arnold et al., 2022), it is relatively rare in eukaryotes (Husnik and McCutcheon, 2018). The reason for this is partly due to the complex cell architecture of eukaryotes, as genetic material must enter the recipient cell’s nucleus to be integrated into the genome (Huang, 2013). Nevertheless, HGT events have been sporadically reported in almost all eukaryotic lineages, including protist (Xiong et al., 2015; Vancaester et al., 2020), fungi (Gonçalves et al., 2018), plant (Wang et al., 2025), and animal (Cummings et al., 2022; Li et al., 2022).

It has been proposed that eukaryotic cell-specific behaviors, including phagocytosis and endosymbiosis, may increase the frequency of HGT (Doolittle, 1998; Margulis et al., 2006). Indeed, genes of the *Wolbachia* bacterial endosymbionts have been found to be horizontally transmitted into the genomes of their insect hosts (Hotopp et al., 2007; Boto, 2014). However, a recent study suggests that any increased opportunity for acquiring new genes through phagocytosis and endosymbiosis is counterbalanced by the reduced need for these genes in eukaryotes, because natural selection in most eukaryotes operates most effectively on genetic variation that is not readily generated by HGT (Keeling, 2024). To gain a more comprehensive understanding of the true phylogenetic and functional scope of HGT-acquired genes in eukaryotes, it’s important to expand the range of investigations to some important but neglected groups, as most of eukaryotic diversity rests within these understudied organisms (Husnik and McCutcheon, 2018).

Ciliates are a diverse clade of unicellular eukaryotes distributed throughout the world, which have occupy different niches and habitats (Jiang et al., 2024). Previous researches have provided insights into how HGT has contributed to the ecological success of these organisms. *Pseudocohnilembus persalinus* is a facultative scuticociliatosis pathogen of mariculture fish. Genomic analyses revealed that *P. persalinus* has acquired numerous prokaryote-derived genes involved in cell adhesion, hemolysis, and heme utilization processes, which may contribute to the virulence of this organism (Xiong et al., 2015). HGT from bacteria has also been linked to the adaptive capabilities of ruminant ciliates. These ciliates inhabit anaerobic, carbohydrate-rich environments, and the acquisition of bacterial genes enables them to more efficiently exploit available resources, thereby enhancing their survival in such specialized ecological niches (Ricard et al., 2006; Feng et al., 2020). Although unicellular, ciliates exhibit phagocytic behavior and endosymbiosis. They are predators of bacteria and feed on them, while also forming a multitude of endosymbiotic associations with different bacteria (Husnik et al., 2021; Fokin and Serra, 2022). However, the extent to which endosymbiotic gene transfer has contributed to the evolution and ecological adaptation of these organisms remains unclear. The genus *Euplotes* is a clade of free-living ciliates. Strikingly, it’s certainly one of the most studied genera for its proneness to establish endosymbiotic relationships with different bacterial organisms (Fokin and Serra, 2022).

*Euplotes* are found throughout every aquatic habitat, from oceans to freshwater bodies to soils (Syberg-Olsen et al., 2016). Like other ciliates, *Euplotes* contains two types of nuclei within one cell: the diploid germline micronucleus (MIC), which enables the transmission of genetic information between generations, but is transcriptionally silent during vegetative growth, and the polyploid somatic macronucleus (MAC), which is transcriptionally active during the vegetative growth (Katz, 2001). Furthermore, a remarkable wealth of symbioses has been observed so far in *Euplotes* (Boscaro et al., 2019). All *Euplotes* species within clade B harbor obligate endosymbiotic bacteria that are essential for host survival and reproduction (Boscaro et al., 2019). However, it remains unclear whether there is HGT between *Euplotes* and the endosymbionts, and how HGT has contributed to *Euplotes* diversification and adaptation. To date, the sole instance of HGT in *Euplotes* is found in *Euplotes raikovi*, which harbor a methionine sulfoxide reductase (Msr) gene likely acquired from a species of Alphaproteobacteria (Dobri et al., 2014).

With advances in genome sequencing technologies, macronuclear genomes of various *Euplotes* species have been sequenced (Wang et al., 2016; Chen et al., 2019; Feng et al., 2022; Jin et al., 2023; Shen et al., 2025), providing valuable resources for systematic investigation of HGT-acquired genes in these ciliates. Here, putative HGT-acquired genes in five sequenced *Euplotes* genomes were detected and the functional bias of HGT-acquired genes were analyzed. And for the first time, we explored the potential endosymbiotic gene transfer events in *Euplotes*. Furthermore, the biological function of the *Ea-ManA* and *Ea-ManB* genes from *E. amieti*, which were transferred into the last common ancestor of *Euplotes*, were studied by qPCR and RNAi.

## 2 Materials and methods

### 2.1 Data sources

The genome and transcriptome data of the *E*. *octocarinatus* were obtained from our previous study (Wang et al., 2016). The publicly available genomes of the other four *Euplotes* species were downloaded from NCBI genome database,^1^ and EvanDB.^2^ For transcriptome data, the raw sequencing reads were downloaded from the NCBI sequence read archive database and then assembled with Trinity v2.3.2 (Grabherr et al., 2011) using the default set of parameters. The detailed information of the data used in this study are listed in Supplementary Table S1. The micronucleus genome (accession number: JAJLLS000000000) of *E*. *woodruffi* was downloaded from Genbank database. *Oxytricha* protein sequences was downloaded from OxyDB.^3^

### 2.2 Gene prediction

Gene prediction were performed by combining *ab initio*, homology, and RNA sequencing prediction methods. The *ab initio* prediction was generated by Augustus, with the assembled transcripts serving as cDNA evidence (Stanke et al., 2006). Protein alignments were generated by aligning *Oxytricha* proteins against the *Euplotes* genome by Miniprot (Li, 2023). Finally, a set of gene models was created using Evidence Modeler (Haas et al., 2008) by weighted combination of evidence from all predicted gene models and RNA-Seq transcripts. BUSCO (Simao et al., 2015) was run using the alveolata_odb10 dataset to estimate the completeness of the predicted gene set.

### 2.3 Identification of bacterial horizontal transferred genes

Horizontally transferred genes in *Euplotes* were identified using a pipeline similar to the approach described by Xiong et al. (2015). In detail, all predicted genes were aligned against the NCBI non-redundant (nr) protein database using BLASTP with an E-value cutoff of 1 × 10^–5^, a parameter also employed in the HGT detection studies of other protist (Xiong et al., 2015; Matriano et al., 2021). We also performed a BLASTX (E-value cut-off = 1 × 10^–5^) search of all *Euplotes* genomes against the nr protein database to identify potential horizontally transferred genes that might be missing from the predicted protein datasets. The genome sequences of symbiotic bacteria are often obtained through metagenomic binning-a method that may inadvertently incorporate host-derived genes into symbiont bins. To prevent the misclassification of these host-origin genes as HGT-acquired gene, a gene was considered a HGT candidate only if its best hit was to a prokaryotic sequence and more than 50% of all hits belonging to prokaryotes. To further distinguish the real HGT-acquired genes from the bacterial contamination, we checked each candidate gene against the following criteria: (i) the corresponding nanochromosome contains *Euplotes*-specific telomeric repeats (C_4_A_4_ or G_4_T_4_), (ii) the transcript contains eukaryote-specific introns. A gene must meet at least one of the two criteria to be retained for further analysis.

Phylogenetic analysis was employed to further validate the candidate HGT genes identified above. Briefly, all candidate genes were subjected to BLASTP search (E-value cut-off = 1 × 10^–5^) against both prokaryotic and eukaryotic protein databases derived from the Refseq data,^4^ to retrieve both eukaryotic and prokaryotic homologs. Proteins with more than five homologs in prokaryotes and no homologs in eukaryotes were identified as horizontal transferred genes (Xiong et al., 2015). If there were homologs in both prokaryotes and eukaryotes, phylogenetic trees were constructed. For phylogenetic analysis, sequences alignments were performed using MUSCLE v3.8.31 with default parameters (Edgar, 2004), and the resulting alignments were used to construct maximum-likelihood trees with FastTree v2.1.11 with the JTT + CAT model (Price et al., 2010), which have been applied in the detection of HGT in the ciliate *P. persalinus* (Xiong et al., 2015). A gene was marked as HGT-acquired gene if it clustered in the prokaryotic clade which had a eukaryotic outgroup.

### 2.4 Genomic feature and expression analysis of HGT-acquired genes

Intron numbers were determined from the predicted gene models and validated by aligning the mRNA sequence with the corresponding genomic sequence using Clustal Omega v1.2.4 with default parameters (Sievers et al., 2011). To determine if there were significant differences between the intron number of candidate HGT-acquired genes compared to other native genes, we performed chi-square (χ^2^) test in R v4.5.1. Coding sequence (CDS) lengths were calculated based on predicted gene models. CDS length distributions of HGT-acquired genes and native genes were compared for each *Euplotes* species using the Wilcoxon rank-sum test to evaluate significant differences. For the *E. woodruffi*, whose MIC genome was available, the distribution of HGT-acquired genes in MIC genome was plotted using R.

The gene expression levels were quantified from RNA-seq data using Salmon v1.10.3 with default parameters (Patro et al., 2017). A transcriptome index was built for each genome, and clean reads were quasi-mapped to the corresponding transcriptome. Gene-level expression abundances were estimated as TPM values. Expression levels of HGT-acquired genes were compared to those of other genes, and statistical differences were assessed using the Wilcoxon rank-sum test.

### 2.5 Functional annotation and subcellular localization analysis of HGT-acquired genes

To characterize the potential functions of HGT-acquired genes in *Euplotes*, functional annotation was performed using multiple databases. Protein sequences of HGT-acquired genes were analyzed using InterProScan to identify conserved protein domains. Gene Ontology (GO) terms were assigned based on InterPro annotations, and Kyoto Encyclopedia of Genes and Genomes (KEGG) pathway annotations were obtained using the KEGG Automatic Annotation Server (KASS) to infer biological pathways associated with HGT-acquired genes (Moriya et al., 2007; Jones et al., 2014).

The subcellular localization of all HGT-acquired proteins was predicted using DeepLoc 2.0., which is a deep learning–based predictor trained on experimentally validated eukaryotic proteins and is suitable for multi-compartment localization inference in non-model eukaryotes (Thumuluri et al., 2022). Predicted localization probabilities were used for downstream statistical summaries and functional enrichment analyses. GO enrichment analysis was performed to identify overrepresented functional categories among HGT-acquired genes in each *Euplotes* species. The analysis was conducted in R using the clusterProfiler package, a widely used tool that provides a statistically robust framework for functional enrichment analysis in comparative genomics studies, with statistical significance assessed by a hypergeometric test (Wu et al., 2021). *P*-values were adjusted for multiple testing using the Benjamini–Hochberg method to control the false discovery rate.

### 2.6 Gene family clustering and phylogenetic analysis of HGT-acquired genes

To investigate the conservation and evolutionary history of HGT-acquired genes among *Euplotes* species, gene family clustering analysis was performed across all five *Euplotes* genomes. Protein sequences of HGT-acquired genes were clustered into gene families based on sequence similarity using OrthoFinder v3.1.1 with default parameters (Emms and Kelly, 2019), which has been used to identify orthologous gene clusters of *Euplotes* species in previous studies (Feng et al., 2022; Jin et al., 2023). Gene families shared among different *Euplotes* species were identified from the clustering results, and families present in all five species were selected for further evolutionary analyses. For each conserved HGT gene family, protein sequences were aligned using MUSCLE v3.8.31 with default parameters. The resulting multiple sequence alignments were used directly for phylogenetic reconstruction. Maximum-likelihood phylogenetic trees were constructed using FastTree v2.1.11 under the JTT + CAT substitution model. Branch support was evaluated based on local support values implemented in FastTree. Phylogenetic trees were visualized and annotated using iTOL (Letunic and Bork, 2024).

### 2.7 Quantitative real-time PCR

*E. amieti* was maintained as monoclonal cultures in freshwater at room temperature. The green alga *Chlorogonium elongatum* was axenically cultured in Synthetic Medium for Chlorogonium (SMC), washed with sterile water, and provided weekly as a food source. Before collection, cells were starved for 5 days to remove algal contamination. Subsequently, cells were collected by centrifugation (4,000 rpm, 5 min). Total RNA (2 μg) was reverse-transcribed into complementary DNA (cDNA) using the TransScript^®^ Uni All-in-One SuperMix for qPCR (TransGen Biotech, Beijing, China). The reverse transcription reaction was performed in a total volume of 20 μL, containing 4 μL of 5 × TransScript^®^ Uni All-in-One SuperMix for qPCR, 1 μL of gDNA Remover, 2 μg of total RNA, and nuclease-free water. The resulting cDNA was used as template for quantitative real-time PCR (qPCR) analysis of *Ea-ManA* and *Ea-ManB*, with *Ea-18S* rRNA serving as the internal reference gene. Gene-specific primers are listed in Supplementary Table S5. All qPCR reactions were performed using PerfectStart^®^ Green qPCR SuperMix (TransGen Biotech, Beijing, China) on an Applied Biosystems 7500 Fast Real-Time PCR system.

### 2.8 RNAi-based gene silencing

RNA interference (RNAi) was employed to suppress the expression of *Ea-ManA* and *Ea-ManB* in *E. amieti* following previously established feeding-based RNAi approaches using bacteria expressing double-stranded RNA (dsRNA) (Xiao et al., 2024). In this system, *E. amieti* cells were fed with engineered bacteria producing gene-specific dsRNA, which induces degradation of the corresponding mRNA in the cytoplasm.

Gene-specific fragments of *Ea-ManA* (738 bp) and *Ea-ManB* (697 bp) were amplified from cDNA by PCR using primers listed in Supplementary Table S5. The amplified fragments were first cloned into the pMD18-T vector (Takara, Japan) and subsequently subcloned into the L4440 vector to generate the RNAi constructs L4440-ManA, L4440-ManB, and L4440-Man (targeting both genes). The resulting plasmids were transformed into the RNase III-deficient *E. coli* strain HT115 for dsRNA production. A 1:100 dilution of overnight bacterial cultures was grown to an OD_600_ of approximately 0.4, after which dsRNA expression was induced by adding 1 mM isopropyl-β-D-1-thiogalactopyranoside (IPTG). Bacterial cultures were further incubated for 4 h until reaching an OD_600_ of approximately 1. The induced bacteria were collected, washed twice with sterile water, and heat-killed at 65°C for 5 min prior to feeding.

Two *E. amieti* cultures were fed in parallel with *E. coli* cells carrying the plasmid to produce Ea-Man-specific dsRNAs or with *E. coli* cells carrying the empty plasmid as control. RNAi feeding experiments were carried out for a total duration of 14 days. Total RNA was extracted on day 7, and knockdown efficiency of *Ea-ManA* and *Ea-ManB* was evaluated by qPCR. The L4440-Man resulted in a 36.8% reduction of *Ea-ManA* and a 46.2% reduction of *Ea-ManB*. Cell numbers were recorded daily throughout the 14-day experimental period to assess the effects of *Ea-Man* gene silencing on cell growth. All experiments were repeated three times independently.

## 3 Results

### 3.1 Detection of HGT candidates in five *Euplotes* species

**FIGURE 1:**
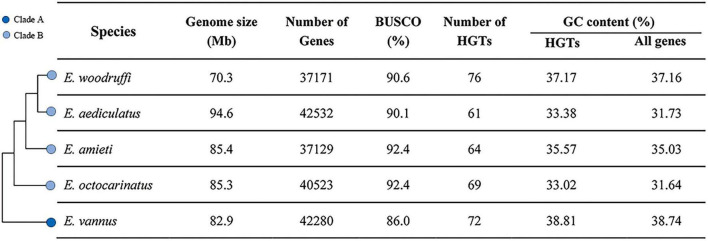
Phylogeny and genomic features of five *Euplotes* species. Phylogenetic tree based on 18S rRNA sequences shows freshwater species (*E. woodruffi*, *E. aediculatus*, *E. amieti*, *E. octocarinatus*) in clade B and marine species (*E. vannus*) in clade A. Genomic features including genome size, number of predicted genes, BUSCO completeness, number of putative HGT-acquired genes, and GC content are summarized.

To systematically identify bacterial-origin HGT events in the genus *Euplotes*, five species with publicly available genomes were collected for comparative analysis: *E. woodruffi*, *E. aediculatus*, *E. amieti*, *E. octocarinatus*, and *E. vannus* (Supplementary Table S1). Among them, only the marine species *E. vannus* was in the clade A, while the other four freshwater species all clustered within clade B (Figure 1). To improve comparability, all genomes were annotated again with the same pipeline. The number of predicted genes in each genome ranged from 37,129 in *E. amieti* to 42,532 in *E. aediculatus*. The completeness of the predicted gene sets was assessed using BUSCO, revealing a relatively high completeness (Figure 1).

Using a conservative phylogeny-based approach (see Materials and methods), 76, 61, 64, 69, and 72 putative HGT-acquired genes were identified in *E. woodruffi*, *E. aediculatus*, *E. amieti*, *E. octocarinatus*, and *E. vannus*, respectively (Supplementary Table S2). The orthologous genes of previously reported horizontally transferred *Msr* gene from *E. raikovi* (Dobri et al., 2014) were also identified in all five *Euplotes* genomes by our pipeline, which validates the robustness of the method we used. To distinguish the HGT-acquired genes from the bacterial contamination, all candidate genes were manually checked. The macronuclear genome of the ciliate *Euplotes* exhibits a distinctive eukaryotic architecture characterized by an abundance of gene-sized DNA molecules (nanochromosomes). These tiny nanochromosomes typically encode single protein coding genes and are flanked by telomeric repeats 5’-(C_4_A_4_)_*n*_-3’ at both ends (Ghosh et al., 1994). We found that about 93% nanochromosomes of the putative HGT-acquired genes contain at least one telomere (Supplementary Table S2). And the remaining telomereless contigs contain transcripts with introns, a typical feature of eukaryotic gene. Furthermore, the GC content of these HGT-acquired genes was found to be similar to the other *Euplotes* genes (Figure 1). Only *E. aediculatus* and *E. octocarinatus* showed significant difference between HGT-acquired genes and native genes regions (*p* < 0.05). Collectively, these results suggest that the HGT-acquired genes identified here are reliable. Detailed information on the putative HGT-acquired genes, including telomere number, length, GC content, tophit and E-value of BLASTP, and functional annotations is presented in Supplementary Table S2.

**FIGURE 2:**
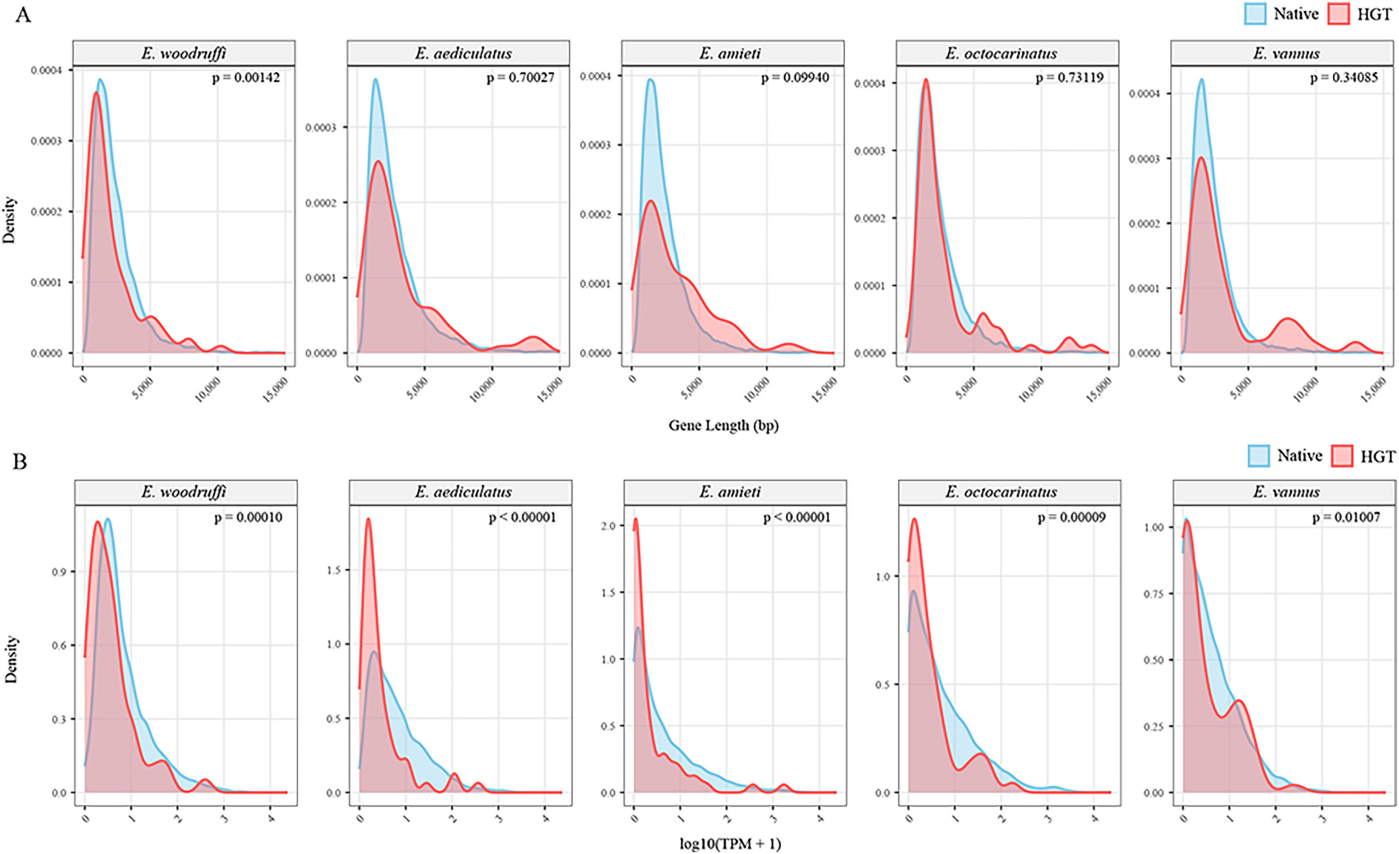
Coding sequence length and gene expression of HGT-acquired genes. **(A)** Density distributions of coding sequence (CDS) lengths for HGT-acquired genes (red) and native genes (blue) in five *Euplotes* species. HGT-acquired genes show significantly shorter CDS lengths only in *E. woodruffi*. **(B)** Density distributions of transcript-level expression for HGT-acquired genes (red) and native genes (blue). HGT-acquired genes exhibit significantly lower expression levels than native genes. Statistical significance was assessed using the Wilcoxon rank-sum test.

Given that introns are a typical eukaryotic gene feature, HGT-acquired genes are expected to have a fewer intron number, especially for recently acquired genes. We found that the proportion of candidate HGTs containing introns was similar to that of other genes in all five *Euplotes* species (Supplementary Figure S1). In addition, we examined the distribution of coding sequence lengths of the candidate HGTs alongside the distribution of coding sequence lengths of other native genes. Results showed no significant difference, except for *E. woodruffi*, which exhibited shorter CDS lengths of HGTS than native genes (Figure 2A). However, the HGT-acquired genes exhibited significantly lower gene expression levels than native genes (Figure 2B).

Although the MAC genome of *Euplotes* is highly fragmented, its MIC genome is composed of megabase-sized chromosomes. Recently, the MIC genome of *E. woodruffi* has been sequenced (Feng et al., 2022), which provides an opportunity to analyze the distribution pattern of HGT-acquired genes on the chromosomes. Of the 76 HGT-acquired genes identified in *E. woodruffi*, 54 were detected in both the MAC and MIC genomes. The 54 HGT-acquired genes were dispersed among 50 different assembled micronuclear scaffolds, only four HGTs were located on two scaffolds (Supplementary Figure S2). Thus, the distribution of HGTs across the MIC genome of *E. woodruffi* shows unbiased.

### 3.2 Possible horizontal gene transfer from the bacterial endosymbionts to *Euplotes*

**FIGURE 3:**
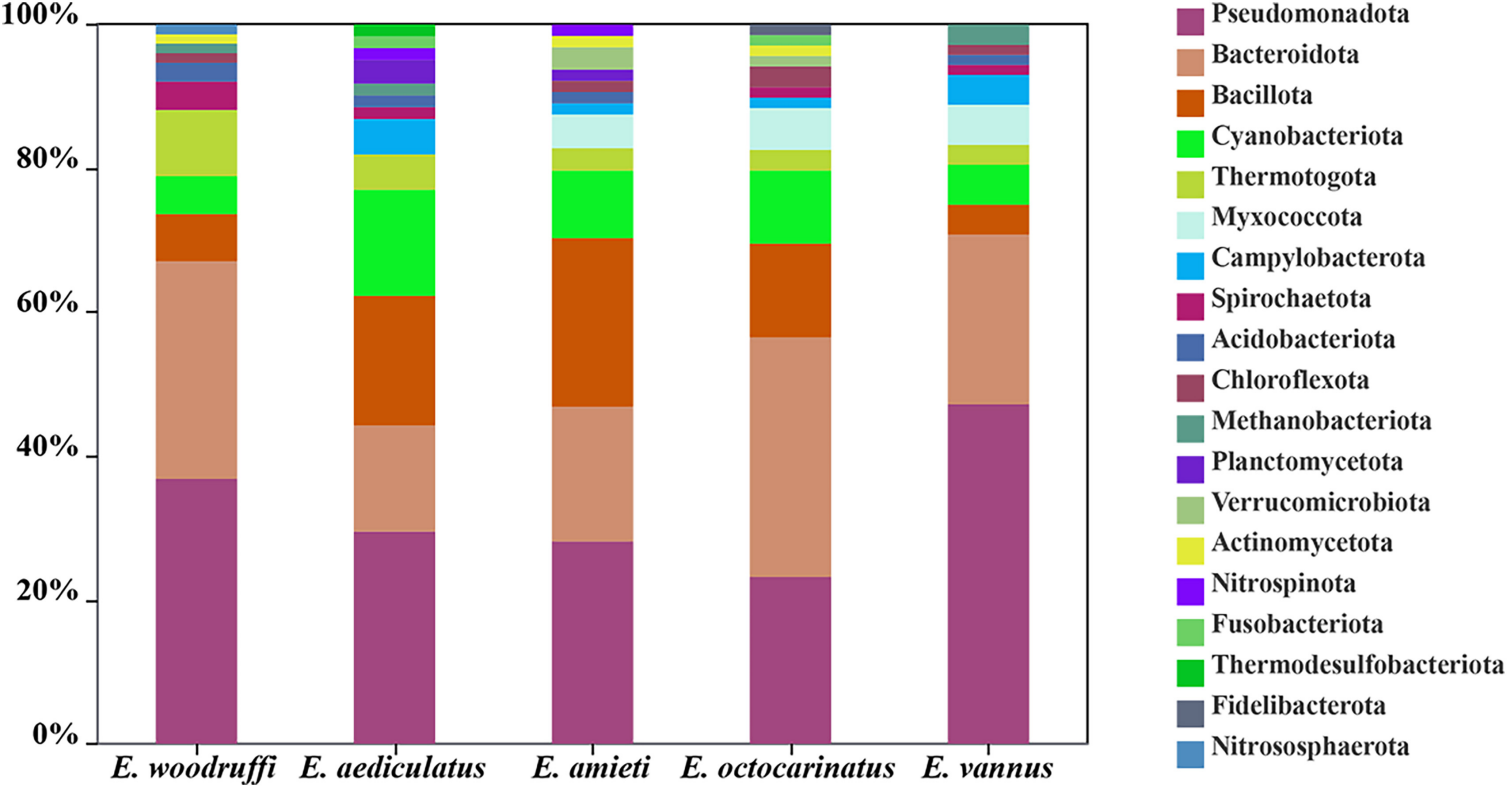
Potential donors of HGT-acquired genes in five *Euplotes* species. Stacked bar plots showing the inferred bacterial donor phyla of horizontally transferred genes in five *Euplotes* species. Each bar represents one *Euplotes* species, and the height of each colored segment indicates the proportion of HGT-acquired genes assigned to a specific bacterial phylum. Colors denote different bacterial phyla, as indicated in the legend.

The potential sources of *Euplotes* HGTs was assessed using the top hit associated with each sequence and associated taxonomic information in the NCBI database. The results suggested that the 342 HGT-acquired genes were likely acquired from 244 putative donor species affiliated to 23 phyla. The most common potential bacterial donors across all five *Euplotes* species belonged to the following phyla: Pseudomonadota, Bacteroidota, Bacillota, and Cyanobacteriota (Figure 3).

**FIGURE 4:**
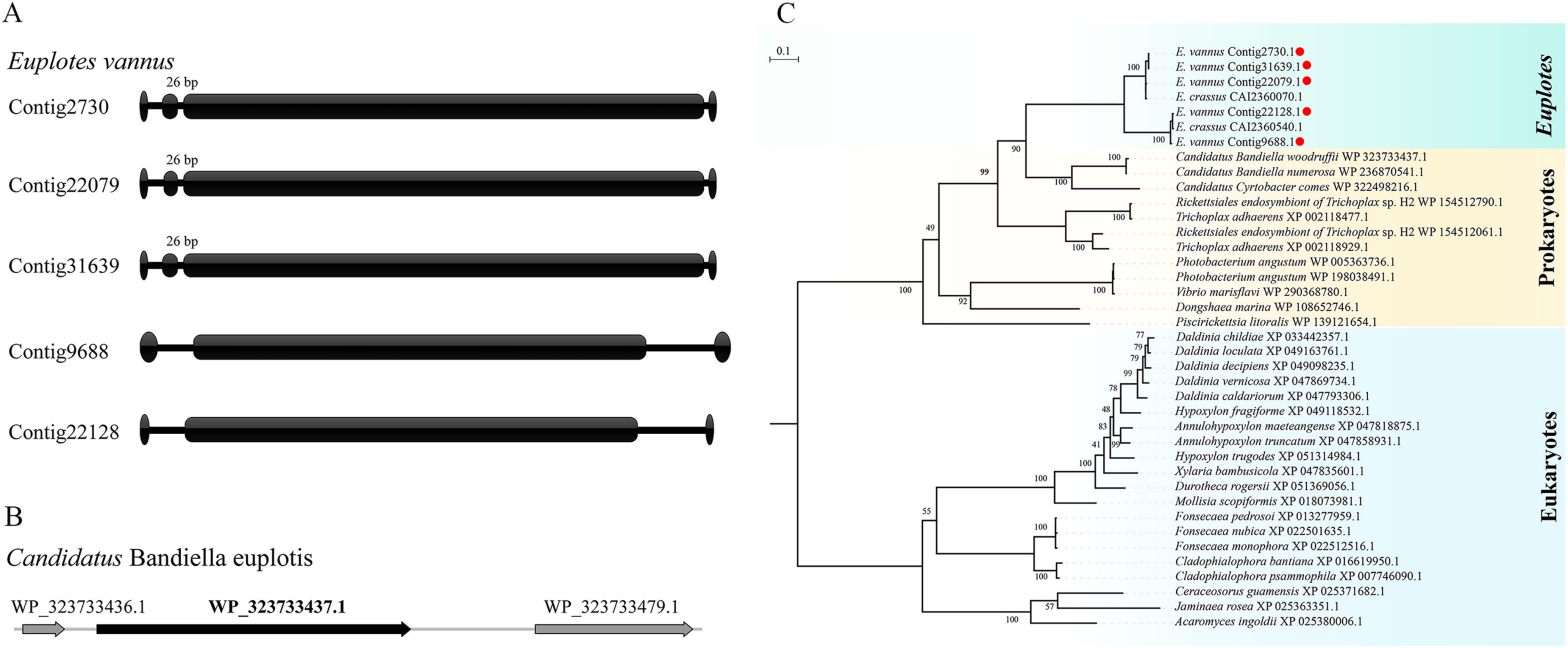
Identification and genomic features of putative endosymbiont-derived HGT genes in *E. vannus*. **(A)** Schematic representation of macronuclear nanochromosomes carrying five putative glycoside hydrolase genes in *E. vannus*. Oval shapes at both ends indicate telomeric repeats, cylinders represent coding sequences (CDSs), and gaps between cylinders denote introns; three of the five genes contain a 26-bp intron. **(B)** Genomic organization of the corresponding loci in *Candidatus Bandiella woodruffii*. Black arrows indicate the glycoside hydrolase gene, and gray arrows represent flanking upstream and downstream genes. **(C)** Maximum-likelihood phylogenetic tree constructed from protein sequences of *Euplotes*, other eukaryotes, and bacteria. Branches are colored according to taxonomic groups: eukaryotes (light blue), bacteria (yellow), and *Euplotes* (light green). The five *E. vannus* genes are indicated by red dots and are positioned within the bacterial clade.

Since some studies have reported that the genes of symbionts have been horizontally transmitted into the host insect genomes (Hotopp et al., 2007; Li et al., 2022), we investigated the association between putative HGT donor organisms and known *Euplotes* endosymbionts. To date, at least 18 genera and 22 species of bacteria have confirmed representatives in *Euplotes* (Supplementary Table S3; Boscaro et al., 2019; Serra et al., 2020; Wang et al., 2023; Wang et al., 2024). Here, a gene is designated as an endosymbiont-derived gene if its putative HGT donor belongs to the same genus as any known *Euplotes* endosymbiont. Finally, we identified five putative endosymbiont-derived genes encoding glycoside hydrolases from *E. vannus.* All sequences share the highest identity (52–54%) with a hypothetical protein (WP_323733437.1) of *Candidatus* Bandiella woodruffii, an accessory symbiont of the *E. woodruffi* (Senra et al., 2016). The nanochromosomes of all five genes contain two telomeres, and three of them contain a 26 bp intron (Figure 4A), suggesting that these five genes belong to *Euplotes*. The genome sequence of *Ca.* Bandiella woodruffii was retrieved from the metagenome assembly (Castelli et al., 2024), which included sequences from both the host *Euplotes* and symbionts. As shown in Figure 4B, the flanking regions of the hypothetical protein encode bacterial homologous proteins, thereby excluding the misassignment of host sequences to the symbiont.

The phylogenetic tree based on protein sequences from *Euplotes* along with other eukaryotes and bacteria suggests the evolutionary relationship of the five genes (Figure 4C). The tree showed that these genes cluster into a monophyletic group among the bacterial clade. The closest bacterial relatives are three midichloriaceae symbionts of *Euplotes* (Giannotti et al., 2022). Furthermore, all seven sequences from *E. vannus* and *E. crassus* clustered into two branches. Therefore, it is likely that ancestral genes were acquired via HGT and subsequently expanded through gene duplication before the divergence of these two *Euplotes* species. Multiple sequence alignment shows a high degree of sequence conservation with the bacteria counterparts (Supplementary Figure S3A). Nevertheless, all *Euplotes* protein sequences is extended by 42 or 50 residues at the N-terminus. Further analysis revealed that the N-terminal extension corresponds to either a signal peptide or transmembrane domain (Supplementary Figure S3B), potentially directing the protein to specific subcellular compartments.

### 3.3 Functions of candidate HGTs in *Euplotes*

**FIGURE 5:**
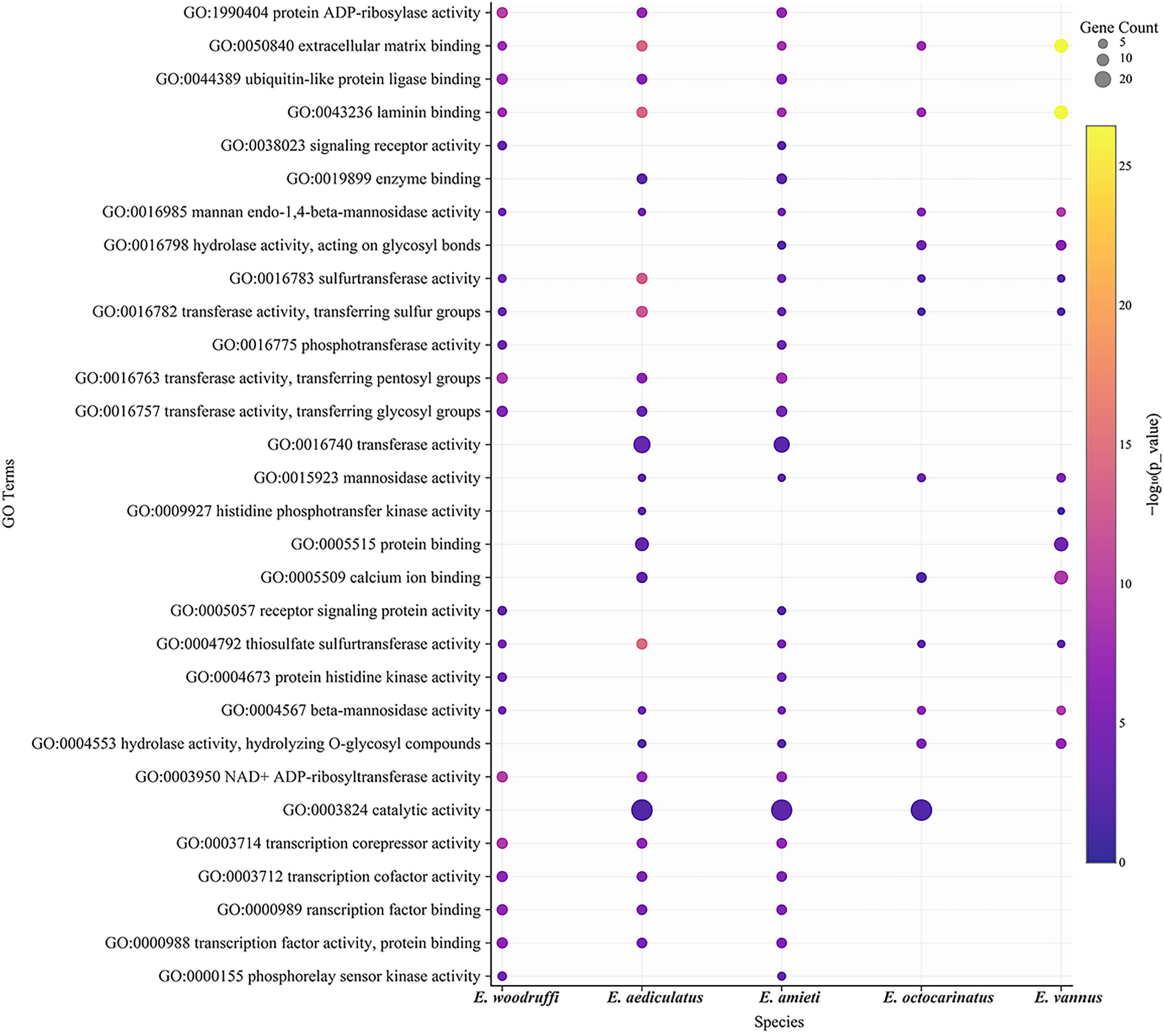
Gene Ontology enrichment analysis of HGT-acquired genes in *Euplotes*. Gene Ontology (GO) enrichment analysis of the 342 HGT-acquired genes across five *Euplotes* species. The dot plot displays the top 30 enriched molecular function GO terms. Each dot represents a GO category; dot size indicates the number of proteins (gene counts) annotated to that term, and dot color reflects the enrichment significance as -log10(*p* value), with darker yellow indicating higher statistical significance. The x-axis represents *Euplotes* species, and the y-axis lists enriched GO terms.

Of the 342 *Euplotes* HGT-acquired genes, 295 (86%) were annotated with a Pfam domain and 270 (79%) with a gene ontology (GO) term (Supplementary Table S2). Moreover, 165 genes were assigned to different KEGG pathways. The GO terms that were enriched for HGT genes in all five *Euplotes* species included mannan endo-1,4-beta-mannosidase activity (GO:0016985), beta-mannosidase activity (GO:0004567), sulfurtransferase activity (GO:0016783), transferase activity, transferring sulfur groups (GO:0016782), thiosulfate sulfurtransferase activity (GO:0004792), extracellular matrix binding (GO:0050840), and laminin binding (GO:0043236) (Figure 5). These results suggested that majority of HGT candidates were enzymes with a variety of catalytic activities.

**FIGURE 6:**
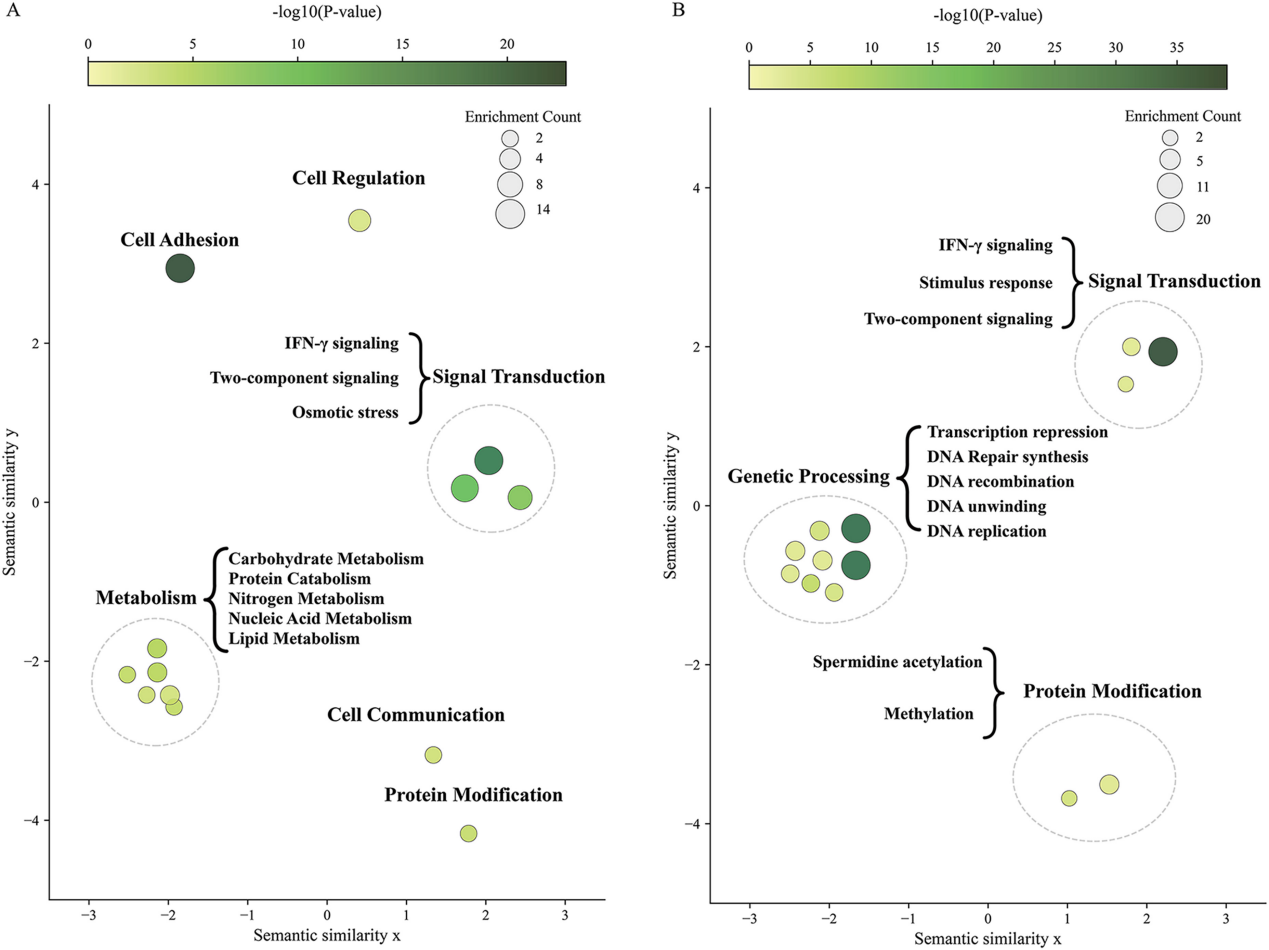
Localization-based functional enrichment of HGT-acquired genes. Scatter plots showing Gene Ontology (GO) biological process terms enriched among cytoplasmic **(A)** and nuclear **(B)** HGT-acquired proteins relative to the complete *Euplotes* proteome. Each circle represents a GO term, with size indicating the number of HGT-acquired proteins annotated to the term and color intensity reflecting enrichment significance (darker green = higher significance).

To understand how these HGT-acquired genes are used, we first examined the subcellular localizations of these proteins to understand where the proteins may operate in host cell. The results suggested that most HGT-acquired genes probably function in the cytoplasm (32–67%) and nucleus (8–33%) (Supplementary Figure S4). To examine the processes that these genes impact in given cellular compartments, we performed localization-based functional enrichments analyses for HGT genes localized in the cytoplasm and nucleus, respectively. The results revealed that cytoplasmic proteins were significantly enriched in metabolism, signal transduction, cell communication, and protein modification (Figure 6A), suggesting that these foreign genes may enhance the host’s metabolic capacity and improve environmental adaptability. Nuclear proteins mainly functioned in genetic information processing, such as DNA replication, repair, recombination, and transcriptional regulation (Figure 6B), implying that these genes may have been functionally integrated into the host’s nuclear regulatory networks, contributing to genome stability maintenance and transcriptional control.

### 3.4 The last common ancestor of *Euplotes* horizontally acquired a foreign gene that involved in the degradation of carbohydrates

**FIGURE 7:**
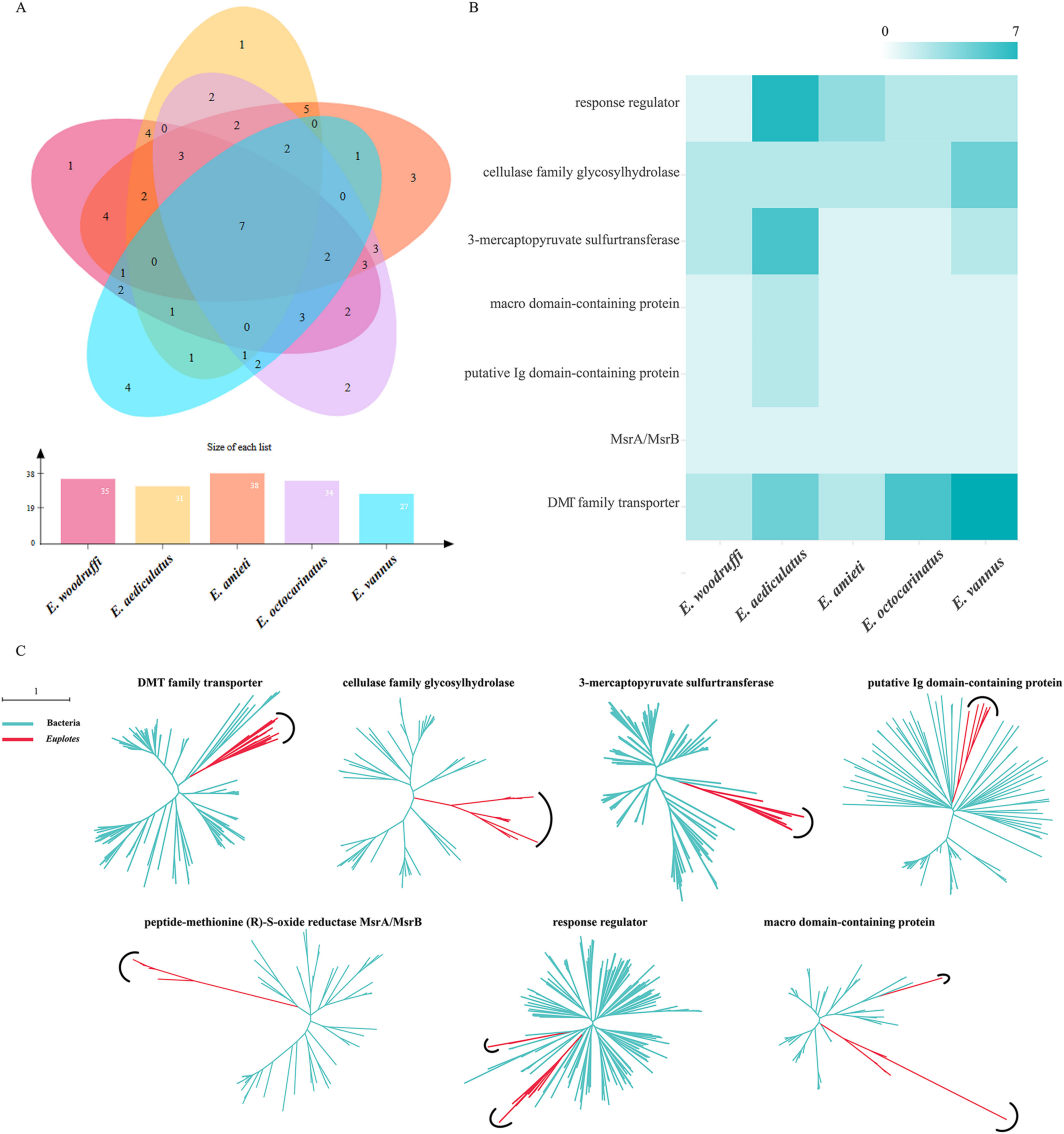
Conservation and evolutionary history of HGT-acquired gene families in *Euplotes*. **(A)** Venn diagram showing HGT-acquired gene families shared among all five *Euplotes* species, together with the number of homologous gene families identified in each species. The bar plot below represents the total number of homologous gene families for each species. **(B)** Heatmap showing the functional classification of the seven conserved HGT families. The x-axis represents species, the y-axis shows functional annotations, and the color intensity reflects the number of genes in each functional category, with darker colors indicating higher counts. **(C)** Maximum-likelihood phylogenetic trees of the seven conserved HGT gene families. Red branches indicate *Euplotes* sequences, and green branches indicate bacterial sequences.

Gene family clustering analysis indicated that only seven HGT families were present in all five *Euplotes* (Figure 7A), including cellulase family glycosylhydrolase, 3-mercaptopyruvate sulfurtransferases, peptide-methionine (R)-S-oxide reductase (MsrA/MsrB), response regulators, macro domain-containing proteins, putative lg domain-containing proteins, and DMT (drug/metabolite transporter) family transporters (Figure 7B). Phylogenetic analysis showed that five gene families (i.e., DMT family transporter, cellulase family glycosylhydrolase, 3-mercaptopyruvate sulfurtransferase, putative Ig domain-containing protein, and peptide-methionine (R)-S-oxide reductase) cluster into a monophyletic (Figure 7C). This result suggests that ancestral genes originated through HGT before the divergence of *Euplotes*. In contrast, the response regulator and macro domain-containing protein gene families showed a different clustering pattern: sequences from the four freshwater species grouped together, while the homologs from the marine species *E. vannus* formed a distinct clade. This divergence may reflect lineage-specific evolutionary trajectories shaped by ecological differentiation. In particular, the divergence between freshwater and marine *Euplotes* suggests that these HGT genes may have undergone adaptive divergence in response to distinct environmental pressures associated with their respective habitats.

We then evaluated the function of the prevalent HGT-acquired genes encoding mannan endo-1,4-β-mannosidase (Man) from *E. amieti*, which was acquired by the last common ancestor of *Euplotes* (Supplementary Figure S5). The macronuclear nanochromosomes carrying the *Ea-ManA* and *Ea-ManB* genes are 1,366 and 2,296 bp long, respectively. The *Ea-ManA* gene is free of introns, and *Ea-ManB* harbors two introns (Supplementary Figure S5A). The *Ea-ManA* gene contains a 1,209 bp open reading frame which encodes a polypeptide of 403 amino acids. The *Ea-ManB* gene contains an ORF of 1,260 bp and the deduced polypeptide consists of 420 amino acids. Both sequences share 47% identity. Phylogenetic analysis showed that these two *Ea-Man* genes cluster into a monophyletic group that is separate from bacterial genes (Supplementary Figure S5B).

**FIGURE 8:**
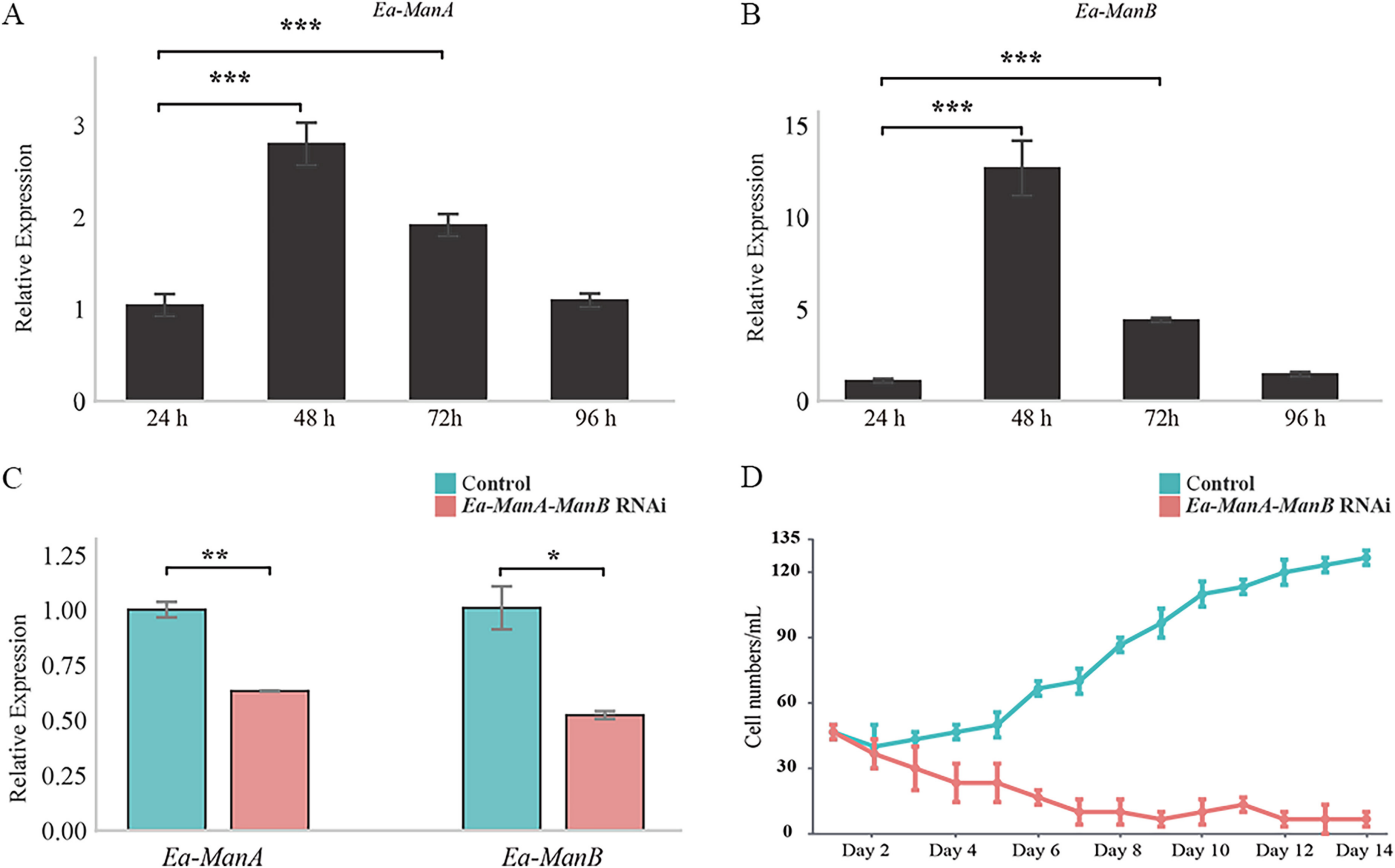
Functional analysis of *Ea-Man* genes in *E. amieti*. **(A,B)** Temporal expression of *Ea-ManA* and *Ea-ManB* analyzed by qPCR at 24, 48, 72, and 96 h after feeding with *Chlorogonium elongatum*, showing peak expression at 48 h. **(C)** qPCR analysis of *Ea-ManA* and *Ea-ManB* transcript levels after RNAi-mediated knockdown. Green bars represent the control group, and red bars represent the RNAi-treated group. **(D)** Growth curves of *E. amieti* cells fed with bacteria carrying the construct of L4440-*Ea-Man* (red) or L4440-Control (green). Data shown are the means ± standard deviations of three biological replicates. *P < 0.05, **P < 0.01, ***P < 0.001.

As *Euplotes* are free-living heterotrophic organisms, we hypothesized that Ea-Man might be involved in food digestion. qPCR analysis was conducted at 24, 48, 72, and 96 h after feeding with *Chlorogonium elongatum*. The results showed that the mRNA levels of *Ea-ManA* and *Ea-ManB* initially increased, then decreased, peaking at 48 h (Figure 8). We further performed a knockdown of *Ea-Man* genes by feeding bacteria containing an expression plasmid encoding dsRNA directed against both *Ea-ManA* and *Ea-ManB* genes. The results suggested that RNAi resulted in a significant knockdown of both *Ea-Man* genes at the level of the transcript (Figure 8C). Daily counting of both cultures were carried out during the 14 days of the experiment. We observed that the cell number was reduced in the specimens fed with bacteria expressing the *Ea-Man*-specific siRNAs compared to the non-silenced cells over the 14 days of bacteria feeding (Figure 8D), showing that Ea-*Man* is essential for growth of *E. amieti*.

## 4 Discussion

While numerous studies have documented HGT events in ciliates, they have predominantly focused on parasitic and symbiotic species (Xiong et al., 2015; Feng et al., 2020; Zhang et al., 2020). In this study, we systematically identified HGT-acquired genes of bacterial origin in five free-living *Euplotes* species, which tend to establish symbiotic relationships with different bacteria, and conducted a comprehensive analysis of their adaptation, origins, and functions.

Our analysis provides evidence of a rich repertoire of HGT events in *Euplotes*, with 61-76 candidate HGT-acquired genes identified across five *Euplotes* species. The number of HGT-acquired genes in *Euplotes* is similar to that in the free-living ciliate *Tetrahymena thermophila* (74 HGT-acquired genes) (Xiong et al., 2015). In contrast, pathogenic ciliates exhibit higher variability: *Pseudocohnilembus persalinus* and *Uronema marinum* contain 54 and 105 HGT-acquired genes, respectively, while *Ichthyophthirius multifiliis* contains only five (Xiong et al., 2015; Zhang et al., 2020). Rumen ciliates demonstrate even greater acquisition of HGT-acquired genes, with transcriptomic analyses revealing 140, 783, and 850 HTGs in *Entodinium furca*, *Diplodinium dentatum*, and *Isotricha intestinalis*, respectively (Feng et al., 2020). Similarly, the colonial choanoflagellate *Salpingoeca rosetta* harbors at least 175 candidate HGT-acquired genes (Matriano et al., 2021). In contrast, a comprehensive survey of HGTs in insects revealed that Lepidoptera, the order with the highest average number of HGT-acquired genes, acquire only 16 genes per species (Li et al., 2022). These findings collectively support the conclusion that unicellular organisms possibly acquire foreign genes more readily than multicellular eukaryotes. This disparity may be due to the physical separation between germline and somatic cells in multicellular eukaryotes, which limits the transmission of foreign genes to offspring.

Recent gene acquisitions are typically characterized by divergent genetic features, including GC content, codon usage bias, and genomic architecture (e.g., intron presence/absence, coding sequence length). Over time, horizontally transferred genes undergo sequence modifications to align with host genome characteristics, thereby enhancing efficiency of transcription and translation (Daubin et al., 2003; Langille et al., 2010). A majority of *Euplotes* HGT candidates had gene features, including GC content, CDS length, and intron content, similar to the rest of the genome (Figure 2), indicating that these acquisitions were not recent. However, the HGT-acquired genes exhibited significantly lower gene expression levels than native genes, suggesting that these genes may have not yet fully adapted to the host’s regulator or their functions have not been sufficiently integrated and utilized.

As heterotrophic organisms, *Euplotes* usually feed on algae and bacteria in natural environments while also forming symbiotic relationships with various bacteria (Boscaro et al., 2019). Our analysis revealed that HGT-acquired genes in *Euplotes* predominantly originated from bacterial groups such as Pseudomonadota, Bacteroidota, Bacillota, and Cyanobacteriota, with very few contributions from obligate intracellular bacteria like *Legionellales*, *Chlamydia*, and *Rickettsiales*. To date, over 18 genera and 22 species of bacteria have been detected in *Euplotes* (Supplementary Table S3). Here, we only identified five putative endosymbiont-derived genes encoding glycoside hydrolases from *E. vannus*. Phylogenetic analyses indicated that these genes most likely originated from a bacterium closely related to the intracellular symbionts *Ca*. Bandiella woodruffii, *Ca*. Bandiella numerosa, and *Ca*. Cyrtobacter comes within the family *Ca.* Midichloriaceae (*Rickettsiales*) (Vannini et al., 2010; Senra et al., 2016; Boscaro et al., 2019). Although these three endosymbionts were all detected in freshwater *Euplotes*, no evidence of HGT from them to the host was found in this study. Metagenomic screening revealed thirteen complete 16S rRNA gene sequences from *E. vannus*, but none belonged to the family *Ca*. Midichloriaceae (Supplementary Table S4). However, *Ca*. Midichloriaceae endosymbionts were also found in many marine hosts (Gruber-Vodicka et al., 2019; Giannotti et al., 2022). It is plausible that *E. vannus* may have harbored transient *Ca*. Midichloriaceae as symbiont or food in the past, but further evidence is required to confirm this hypothesis. These results suggested that HGT from endosymbionts to *Euplotes* is possible, albeit occurring at a relatively low frequency. A similar observation was described in an analysis of the genomes and transcriptomes of marine euglenozoan flagellates of genus *Rhynchopus* which also revealed that there is no evidence of endosymbiotic gene transfer in symbiont-bearing diplonemids (Valéria et al., 2026). Thus, although the ability to engulf bacteria and harbor various endosymbionts indeed expose the cell to more foreign DNA, there is no evidence that these eukaryotic genomes have been continuously augmented by genes derived from prey or symbionts. This observation may support the view that any increased potential for acquiring new genes through phagocytosis and endosymbiosis is counterbalanced by a reduced need for these genes in eukaryotes, as natural selection in most eukaryotic lineages primarily acts on variation not readily generated by HGT (Keeling, 2024).

The genus *Euplotes* is prone to establishing endosymbiotic relationships with diverse bacterial organisms (Fokin and Serra, 2022). Notably, all *Euplotes* species within clade B harbor obligate endosymbiont that are essential for host survival and reproduction (Boscaro et al., 2019). However, our results suggested no evidence of HGT from these endosymbionts in all four *Euplotes* species examined within clade B. Previous studies have reported that while the genomes of some protists and insects with obligate endosymbionts encode a few proteins targeted to their symbionts, none of these genes are derived from the same bacterial lineage as the endosymbiont itself (Morales et al., 2023; Husnik et al., 2013). The absence of such transfers suggests that HGT or at least the stable retention of HGT-acquired genes is not essential for successful endosymbiosis in *Euplotes*. Given that the genomes of these essential symbionts typically undergo substantial reduction in size and coding capacity (Wang et al., 2024; Boscaro et al., 2022), it’s worth to investigating whether these HGT-acquired genes can also encoding proteins targeted to the symbionts in *Euplotes*.

According to the hypothesis proposed by Jain et al. (1999), the transferability of genes depends on two key factors: gene function and the extent of protein-protein interactions. Operational genes, which can function independently of other genes, are more likely to undergo HGT. In contrast, informational genes can fas those involved in transcription and translation—typically interact physically with a greater number of gene products. This interdependence significantly limits their functionality when transferred individually, thereby reducing the likelihood of successful retention as horizontally transferred genes. In this study, we found that most HGT-acquired genes with known functions in *Euplotes* were orthologous to operational genes, such as enzymes that function in carbohydrate metabolism and sulfur metabolism, as well as genes that function in signal transduction. Furthermore, we identified seven conserved ancestral gene families in *Euplotes*. Functional annotation revealed that these conserved genes encode proteins including glycoside hydrolases, sulfurtransferases, methionine sulfoxide reductases, response regulators, macro domain-containing proteins, putative immunoglobulin domain-containing proteins, and drug/metabolite transporters. This suggested their important roles in the environmental adaptation and metabolic regulation of the host. Interestingly, horizontal gene transfer in diverse protists often involves enzymes that function in carbohydrate metabolism (Feng et al., 2020; Vancaester et al., 2020; Matriano et al., 2021). *Euplotes* also possesses multiple glycosyl hydrolases and glycosyltransferases that were likely acquired through HGT. The conservation of horizontally acquired glycosyl hydrolases in *Euplotes* suggests their function importance in digesting food sources. Supporting this, qPCR analysis revealed that the mRNA levels of *Ea-ManA* and *Ea-ManB* initially increased and subsequently decreased after feeding with *C. elongatum* in *E. amieti*. Furthermore, knockdown of *Ea-Man* genes led to increased mortality of *E. amieti*, reinforcing their critical role in survival.

## 5 Conclusion

In this study, genome-wide analyses of five *Euplotes* species revealed extensive bacterial-derived HGT events, which exhibited both conserved and species-specific patterns. Most of these genes were likely acquired through ancient HGT events, as evidenced by similar genomic signatures to the host. Phylogenetic analysis indicated that the majority of HGTs originated from dominant environmental bacteria, while a subset may have been derived from intracellular symbionts, highlighting multiple pathways for the acquisition of foreign genes. Functional predictions demonstrated that these horizontally transferred genes are broadly involved in signal transduction, metabolic regulation, and stress-response processes, suggesting their potential roles in enabling *Euplotes* to adapt to environmental changes and maintain physiological homeostasis. Finally, functional validation of mannan endo-1,4-β-mannosidase genes in *E. amieti* provided evidence that HGT-derived genes contribute to food digestion, thereby enhancing digestive capacity and ecological fitness.

## Data Availability

The original contributions presented in the study are included in the article/Supplementary material, further inquiries can be directed to the corresponding author.

## Footnotes

1
https://www.ncbi.nlm.nih.gov/genome

2
https://evan.ciliate.org/index.php/home/index.php

3
https://oxy.ciliate.org/index.php/home/index.php

4
https://ftp.ncbi.nih.gov/refseq/release/

## Supplementary material

Supplementary Figure S1 Intron content of HGT-acquired and native genes in five *Euplotes* species. The proportions of genes with and without introns are shown for HGT-acquired genes and native genes in *E. woodruffi*, *E. aediculatus*, *E. amieti*, *E. octocarinatus*, and *E. vannus*. Green indicates genes containing introns, and gray indicates intronless genes. No significant difference in intron content was detected between HGT-acquired and native genes. Statistical significance was assessed using the chi-square (χ^2^) test.

Supplementary Figure S2 Distribution of HGT-acquired genes on the micronuclear (MIC) genome of *E. woodruffi*. Among the 76 HGT-acquired genes identified in the macronuclear (MAC) genome, 54 were also detected in the MIC genome. These 54 genes are dispersed across 50 MIC scaffolds, with only four genes located on two scaffolds. The overall distribution of HGT-acquired genes across the MIC genome appears unbiased, showing no obvious clustering on specific scaffolds. Green indicates MIC scaffolds harboring a single MAC-derived HGT gene, whereas orange indicates MIC scaffolds containing more than one MAC-derived HGT gene.

Supplementary Figure S3 Sequence conservation and N-terminal extensions of putative endosymbiont-derived genes. **(A)** Multiple sequence alignment showing a high degree of conservation between *Euplotes* proteins and their bacterial counterparts. The red box highlights the N-terminal extensions present in *Euplotes* sequences but absent in bacterial homologs. **(B)** The N-terminal extensions, consisting of 42–50 amino acid residues, are predicted to form signal peptides (red) or transmembrane domains (yellow), suggesting a potential role in directing subcellular localization of these proteins.

Supplementary Figure S4 Subcellular localization of HGT-acquired proteins in *Euplotes*. Predicted subcellular localizations of HGT-acquired proteins in five *Euplotes* species. Most HGT proteins are predicted to function in the cytoplasm (32%–67%) and nucleus (8%–33%). Each species is represented by a different colored circle, and smaller circles beneath each species indicate the proportion of HGT proteins assigned to different subcellular compartments.

Supplementary Figure S5 Characterization of *Ea-Man* genes in *E. amieti*. **(A)** Schematic representation of *Ea-ManA* and *Ea-ManB* on macronuclear nanochromosomes. Ellipses at both ends indicate telomeres; cylinders represent coding sequences (CDS), and the gaps between cylinders indicate introns. *Ea-ManA* is intronless, whereas *Ea-ManB* contains two introns of 27 and 677 bp. **(B)** Maximum-likelihood phylogenetic tree of *Ea-ManA* and *Ea-ManB* protein sequences with homologs from other eukaryotes and bacteria. Branch colors indicate taxonomic groups: *Euplotes* (light green), other eukaryotes (light blue), and bacteria (yellow), with *Euplotes* sequences clustering within the bacterial clade.
