# Supplementary material for: Detection and functional analysis of horizontal gene transfer events in the ciliate Euplotes
Source: Front Microbiol. 2026 Apr 8;17:1782463. doi: 10.3389/fmicb.2026.1782463 (PMC13071891; doi:10.3389/fmicb.2026.1782463)
Supplement: Supplementary file 1 [file Table_1.docx]

**Table S1. Overview of *Euplotes* species and the data source used in this study.**

| **Species** | **Genome data** | **Transcriptome data** | **Habitat type** | **Reference** |
| --- | --- | --- | --- | --- |
| *E. octocarinatus* | EOGD (http://ciliates.ihb.ac.cn/database/home/#eo) | SRA (SRX1270740) | Freshwater | Ruanlin Wang *et al*. Scientific Reports, 2016 |
| *E. amieti* | GenBank (GCA_048569255.1) | \ | Freshwater | Liheng Sheng *et al*. Frontiers in Microbiology, 2025 |
| *E. woodruffi* | GenBank (GCA_027382605.1) | SRA (SRR21815378, SRR21815379, SRR21815380) | Freshwater | Yi Feng *et al*. eLife, 2022 |
| *E. aediculatus* | GenBank (GCA_030463445.1) | SRA (SRR22331104) | Freshwater | Didi Jin *et al*. Marine Life Science & Technology, 2023 |
| *E. vannus* | EvanDB (https://evan.ciliate.org/index.php/home/index.php) | SRA (SRR1395674, SRR13956742) | Marine | Xiao Chen *et al*, Molecular ecology resources, 2019 |
