# Supplementary material for: Detection and functional analysis of horizontal gene transfer events in the ciliate Euplotes
Source: Front Microbiol. 2026 Apr 8;17:1782463. doi: 10.3389/fmicb.2026.1782463 (PMC13071891; doi:10.3389/fmicb.2026.1782463)
Supplement: Supplementary file 5 [file Table_5.docx]

| **Primers** | **Sequence(5′ → 3′)** |
| --- | --- |
| Primers for construction of interference plasmids | |
| Ea-ManA-F | TCCCCGCGGGACCTGGACGATATGAAGA |
| Ea-ManA-R | TGCTCTAGACTCTTCAAGGACCATTGG |
| Ea-ManB-F | TGCTCTAGAGACAGAGAAGGATTGTACC |
| Ea-ManB-R | CCGCTCGAGGGATCATATGATCCATAGTCT |
| Primers for qPCR | |
| Ea-ManA-F1 | TGGGCTTATGGAGGAGAAGGAAG |
| Ea-ManA-R1 | CTTGTCTTTCATGGGGAGGATCTC |
| Ea-ManB-F1 | CGACCCTCCACATGAAAGACAAG |
| Ea-ManB-R1 | TTCCGACTTGACTAATTTGCTTTGC |
| Ea-18SrRNA-F | ACATAGCGAGGATTGACAGATTGATAG |
| Ea-18SrRNA-R | ACGAATCACTCCACGAACTAAGAAC |

**Table S5 List of primers used in this study.**
