# Supplementary figures and images for: Detection and functional analysis of horizontal gene transfer events in the ciliate Euplotes
Source: Front Microbiol. 2026 Apr 8;17:1782463. doi: 10.3389/fmicb.2026.1782463 (PMC13071891; doi:10.3389/fmicb.2026.1782463)

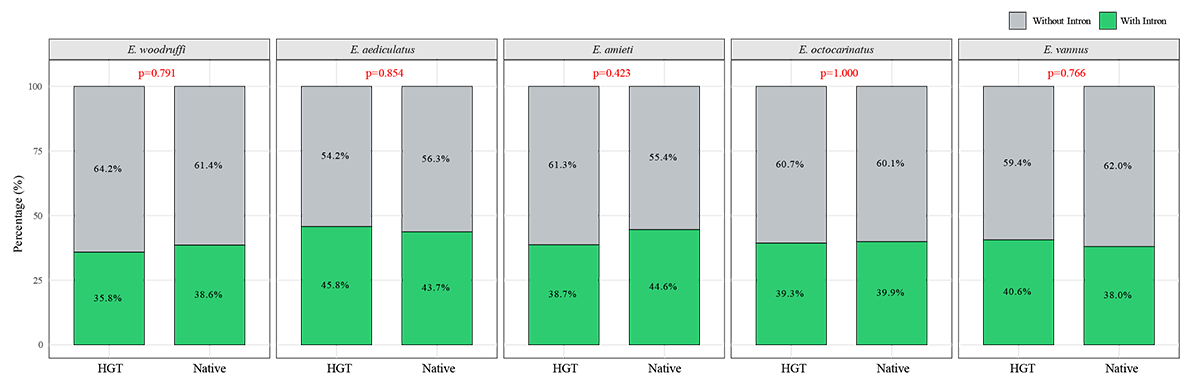

Supplement: Supplementary Figure S1 — Intron content of HGT-acquired and native genes in five Euplotes species. The proportions of genes with and without introns are shown for HGT-acquired genes and native genes in E. woodruffi, E. aediculatus, E. amieti, E. octocarinatus, and E. vannus. Green indicates genes containing introns, and gray indicates intronless genes. No significant difference in intron content was detected between HGT-acquired and native genes. Statistical significance was assessed using the chi-square (χ2) test. [file Image_1.tif]

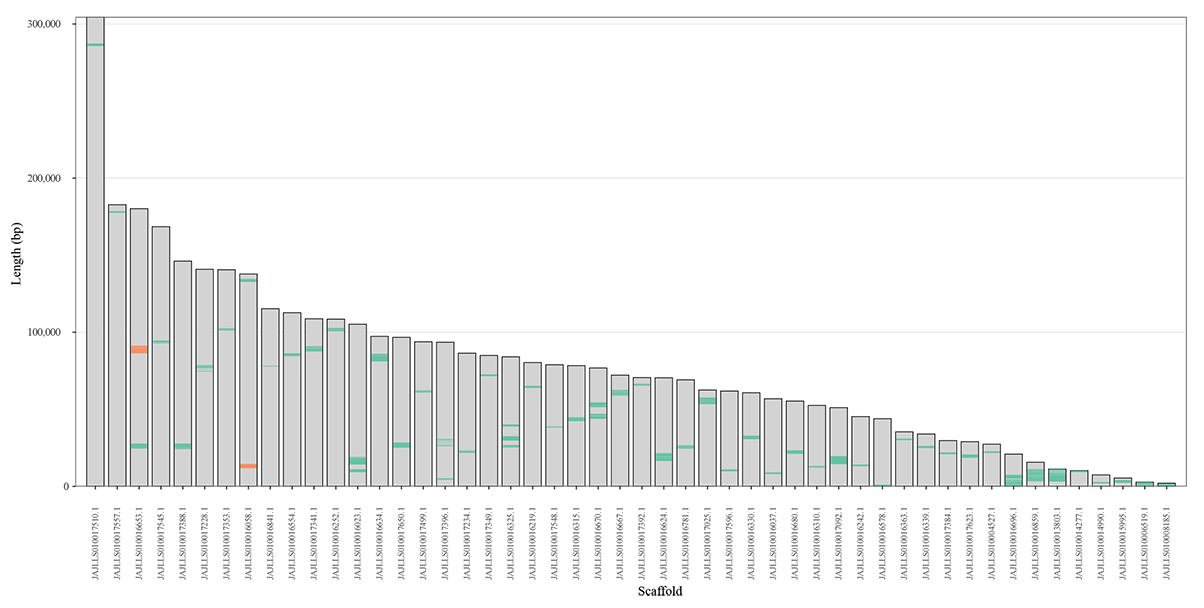

Supplement: Supplementary Figure S2 — Distribution of HGT-acquired genes on the micronuclear (MIC) genome of E. woodruffi. Among the 76 HGT-acquired genes identified in the macronuclear (MAC) genome, 54 were also detected in the MIC genome. These 54 genes are dispersed across 50 MIC scaffolds, with only four genes located on two scaffolds. The overall distribution of HGT-acquired genes across the MIC genome appears unbiased, showing no obvious clustering on specific scaffolds. Green indicates MIC scaffolds harboring a single MAC-derived HGT gene, whereas orange indicates MIC scaffolds containing more than one MAC-derived HGT gene. [file Image_2.tif]

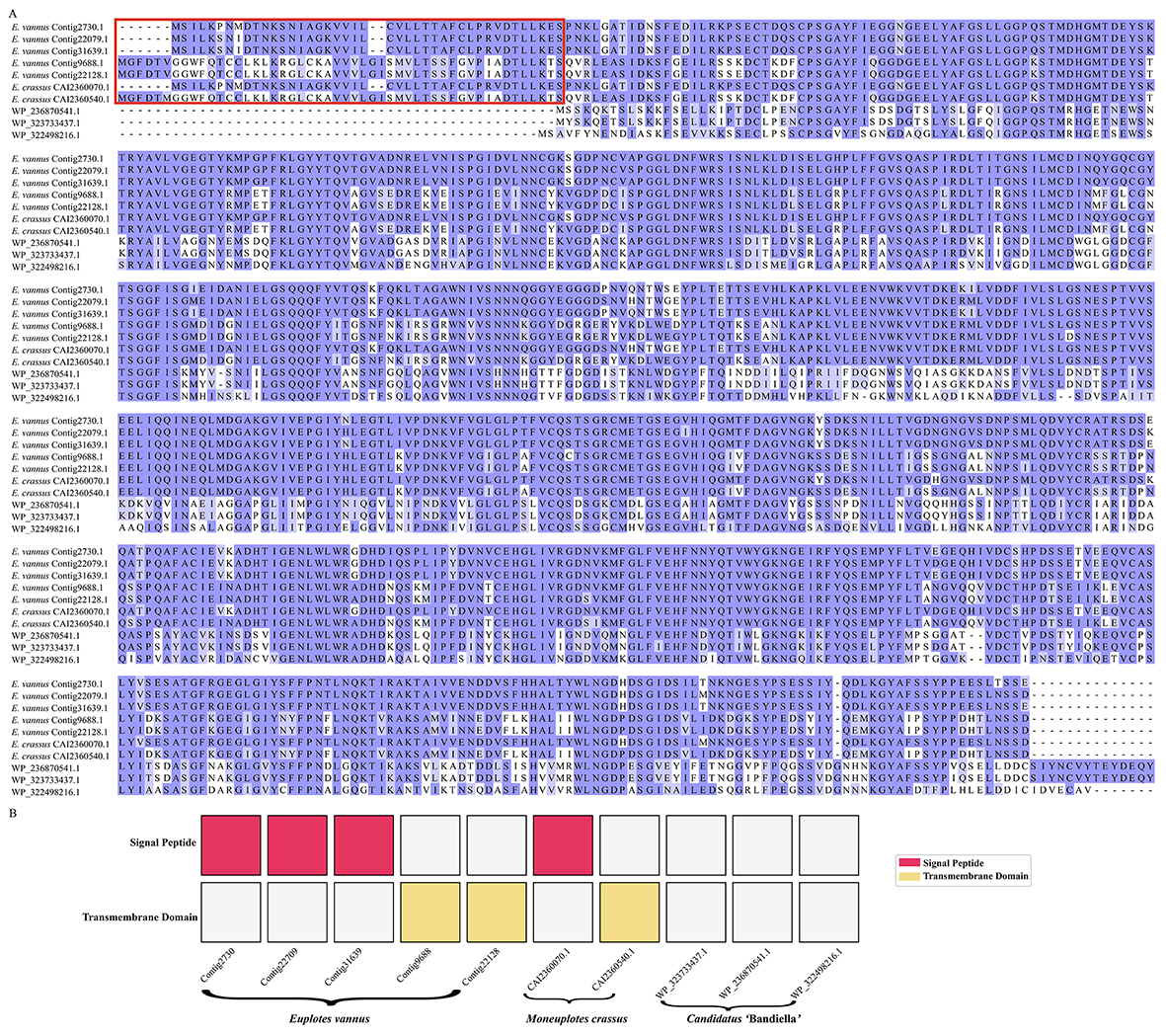

Supplement: Supplementary Figure S3 — Sequence conservation and N-terminal extensions of putative endosymbiont-derived genes. (A) Multiple sequence alignment showing a high degree of conservation between Euplotes proteins and their bacterial counterparts. The red box highlights the N-terminal extensions present in Euplotes sequences but absent in bacterial homologs. (B) The N-terminal extensions, consisting of 42–50 amino acid residues, are predicted to form signal peptides (red) or transmembrane domains (yellow), suggesting a potential role in directing subcellular localization of these proteins. [file Image_3.tif]

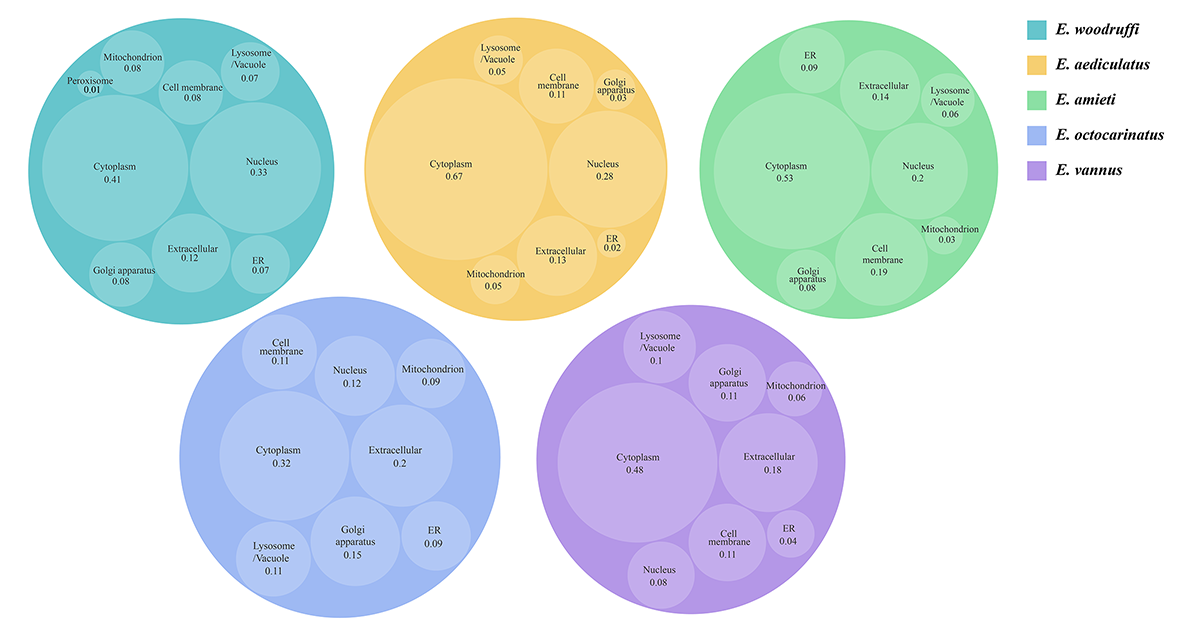

Supplement: Supplementary Figure S4 — Subcellular localization of HGT-acquired proteins in Euplotes. Predicted subcellular localizations of HGT-acquired proteins in five Euplotes species. Most HGT proteins are predicted to function in the cytoplasm (32%–67%) and nucleus (8%–33%). Each species is represented by a different colored circle, and smaller circles beneath each species indicate the proportion of HGT proteins assigned to different subcellular compartments. [file Image_4.tif]

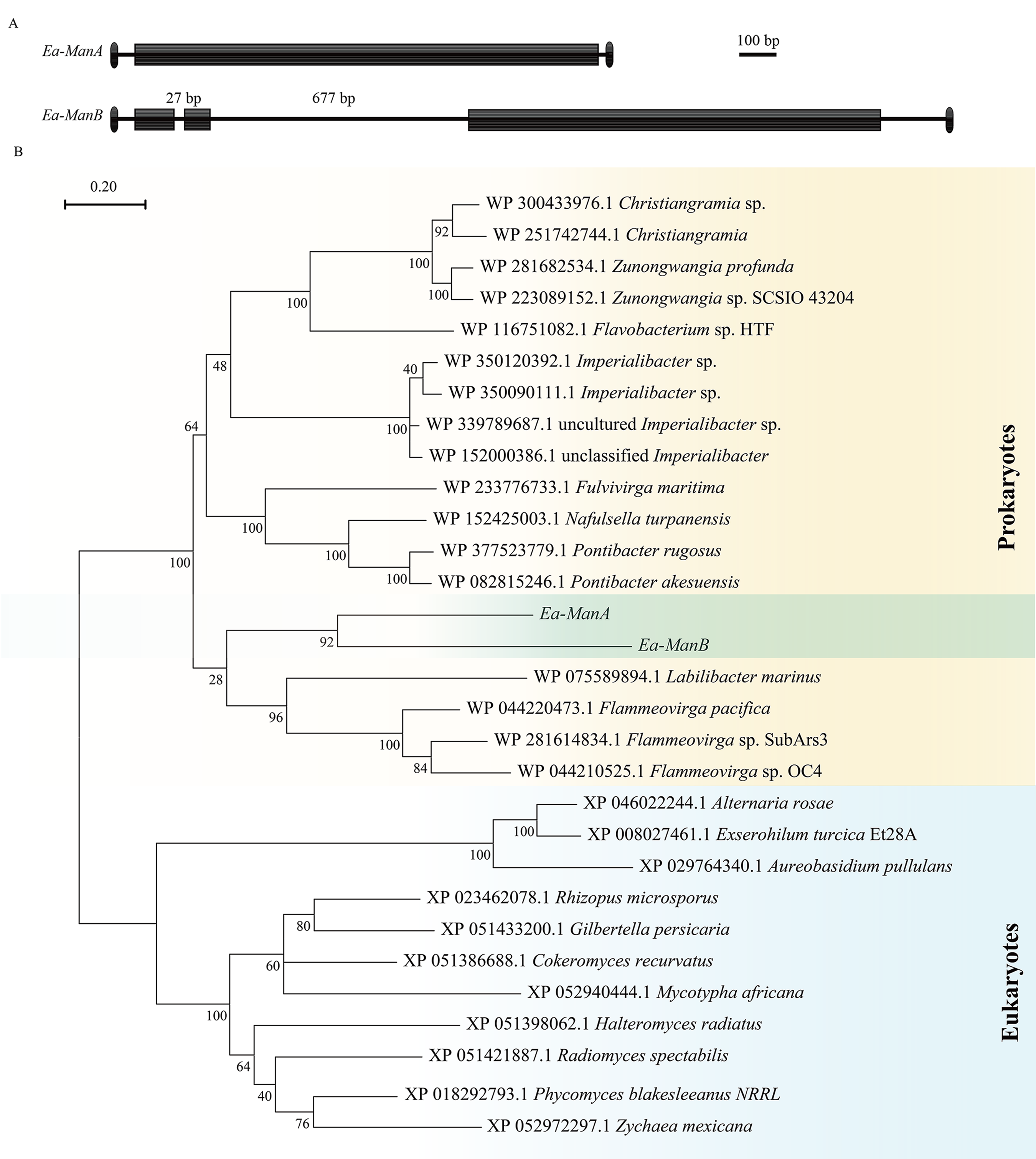

Supplement: Supplementary Figure S5 — Characterization of Ea-Man genes in E. amieti. (A) Schematic representation of Ea-ManA and Ea-ManB on macronuclear nanochromosomes. Ellipses at both ends indicate telomeres; cylinders represent coding sequences (CDS), and the gaps between cylinders indicate introns. Ea-ManA is intronless, whereas Ea-ManB contains two introns of 27 and 677 bp. (B) Maximum-likelihood phylogenetic tree of Ea-ManA and Ea-ManB protein sequences with homologs from other eukaryotes and bacteria. Branch colors indicate taxonomic groups: Euplotes (light green), other eukaryotes (light blue), and bacteria (yellow), with Euplotes sequences clustering within the bacterial clade. [file Image_5.tif]
